# Salivary and serum androgens with anti-Müllerian hormone measurement for the diagnosis of polycystic ovary syndrome

**DOI:** 10.1038/s41598-018-22176-1

**Published:** 2018-02-28

**Authors:** Thozhukat Sathyapalan, Ahmed Al-Qaissi, Eric S. Kilpatrick, Soha R. Dargham, Brian Keevil, Stephen L. Atkin

**Affiliations:** 1 Department of Diabetes and Endocrinology, University of Hull, Hull, UK; 2 Division of Pathology, Sidra Research Centre, Doha, Qatar; 3 Infectious Disease Epidemiology Group, Weill Cornell Medicine, Doha, Qatar; 4 Department of Clinical Biochemistry, Wythenshawe Hospital, Manchester, UK; 5 Research Faculty, Weill Cornell Medicine, Doha, Qatar

## Abstract

To determine the predictive value of a raised androgen level with an elevated anti-Müllerian hormone (AMH) for the diagnosis or exclusion of polycystic ovary syndrome (PCOS), a prospective cross-sectional study of 170 women (105 with PCOS type A and 65 normal) was undertaken. AMH was combined with one of, total serum testosterone (T); calculated free androgen index; salivary testosterone (salT); serum androstenedione (A); salivary androstenedione (salA). The diagnostic sensitivity and specificity of AMH (>35 pmol/l) alone for PCOS were 55% and 79% respectively. The diagnostic sensitivity and specificity of AMH (>35 pmol/l) with either an elevated T or raised FAI level for PCOS showed 100% specificity and a 100% positive predictive value. Conversely, diagnostic exclusion of PCOS was shown by an AMH <35 pmol/l with a normal T or FAI salivary testosterone giving 100% specificity and 100% positive predictive value. AMH with an elevated A or elevated salA level gave specificities of 87% and 94%, and positive predictive values 80% and 94%, respectively. Therefore, the combination of an AMH with a cut off of 35 pmol/l combined with a raised T and/or a FAI will confirm PCOS whilst a normal AMH with a normal T and/or FAI will exclude PCOS, thus addressing diagnostic uncertainty.

## Introduction

Polycystic ovary syndrome (PCOS) is the most common endocrine disorder affecting women of reproductive age and its prevalence varies according to the criteria used between 6% (NIH criteria) and 10% (Androgen Excess Society or Rotterdam society guidelines)^1^; however, it still remains a diagnosis only after the exclusion of other conditions^2,3^. Anti-Müllerian hormone (AMH) is produced in the granulosa cells by the pre-antral and small antral follicles and highly correlates to the antral follicle count. The antral follicle count is increased in PCOS and hence PCOS is associated with higher serum AMH values^4^; higher AMH levels are particularly associated with PCOS patients fulfilling all three diagnostic criteria^5^. It has been suggested that AMH may add value for the positive diagnosis of PCOS, with a meta-analysis suggesting a serum AMH cut-off level of 35 pmol/l that has a sensitivity of 79.4% and a specificity of 82.8%^6^. We have recently reported that the combination of a raised FAI with a raised salT identified 100% of PCOS patients in a biobank cohort, thought this was not found with serum A or salA^7^, though others have suggested that serum A reflects androgen excess in PCOS^8^ and has diagnostic value^9^. Therefore, the aim of this study was to look specifically if AMH would be complementary for the diagnosis of PCOS in combination with an elevated androgen levels (T or FAI or salT or A or salA) within this well-defined cohort of PCOS patients from the general population that fulfilled all 3 of the Rotterdam criteria.

## Subjects and Methods

This was a cross sectional study involving 105 well characterised women with PCOS and 65 women without PCOS who presented sequentially and prospectively to the Department of Endocrinology and were recruited to the local PCOS biobank (ISRCTN70196169). All patients gave written informed consent. This study was approved by the Newcastle & North Tyneside Ethics committee. The diagnosis of PCOS was based on all three diagnostic criteria of the Rotterdam consensus, namely clinical and biochemical evidence of hyperandrogenism (Ferriman-Gallwey score >8; free androgen index >4, total testosterone >1.5 nmol/l), oligomenorrhea or amenorrhea and polycystic ovaries on transvaginal ultrasound^10^, described as the type A phenotype in which AMH levels are reported to be highest^5^. Study participants had no concurrent illness, were not on any medication for the preceding 9 months and were not planning to conceive. Non-classical 21-hydroxylase deficiency, hyperprolactinemia, Cushing’s disease and androgen-secreting tumours were excluded by appropriate tests. All of the control women had regular periods, no clinical or biochemical hyperandrogenism, no polycystic ovaries on ultrasound, no significant background medical history and none of them were on any medications including oral contraceptive pills or over the counter medications. All women underwent a 75 g oral glucose tolerance test to exclude impaired glucose tolerance and type 2 diabetes. All women with PCOS and control women were Caucasian. Height, weight, waist circumference and body mass index (BMI) were performed according to WHO guidelines^11^.

### Study measurements

Blood samples were centrifuged within 5 min of collection and were stored frozen at −80 °C pending analysis. All study measurements and analysis were performed in accordance with the relevant guidelines and regulations. Serum T and A were measured by LC/MS/MS on an Acquity UPLC system coupled to a Quattro Premier XE mass spectrometer (Waters, Manchester, UK). Sex hormone binding globulin (SHBG) was measured by an immunometric assay with fluorescence detection on the DPC Immulite 2000 analyzer using the manufacturer’s recommended protocol (upper limit of the reference range 2.0 nmol/l). The free androgen index (FAI) was calculated as the total testosterone × 100/SHBG. Serum insulin was assayed using a competitive chemiluminescent immunoassay performed on the manufacturer’s DPC Immulite 2000 analyzer (Euro/DPC, Llanberis, UK). The analytical sensitivity of the insulin assay was 2 μU/ml, the coefficient of variation was 6%, and there was no stated cross-reactivity with proinsulin. Plasma glucose was measured using a Synchron LX 20 analyzer (Beckman-Coulter), using the manufacturer’s recommended protocol. The coefficient of variation for the assay was 1.2% at a mean glucose value of 5.3 mmol/liter. The insulin resistance was calculated using the HOMA method [HOMA-IR = (insulin × glucose)/22.5]. Anti-Müllerian hormone was measured using a Beckman Coulter Access automated immunoassay; between run precision was <3% across the range measured.

### Collection and handling of saliva samples

This has been detailed previously for the saliva collection and for the salivary androgen measurement methodology^7^. In brief, participants were asked to spit or drool directly into a 4 mL sealable polystyrene tube and to provide at least 3 mL of saliva. Unstimulated saliva samples were used to avoid any assay interference. The “passive drool” technique was used for the collection of saliva rather than the ‘salivette’ method. Salivary testosterone and salivary androstendione were measured by LC-MS/MS analysis performed using a Waters Acquity UPLC system coupled to a Waters Xevo TQS mass spectrometer, giving a lower limit of quantification of 5 pmol/L for salT and 6.25 pmol/l for salA with an inter and intra-assay precision coefficient of variation of <4% and <7.5%, respectively^7^.

### Statistical analysis

Data trends were visually evaluated for AMH and for each androgen, and non-parametric tests were applied on data that violated the assumptions of normality when tested using Kolmogorov-Smirnov Test. Accordingly, comparative analysis evaluating androgen levels between PCOS cases and controls were performed using the non-parametric Mann-Whitney tests. Significance was defined at α = 0.05. All analyses were done using IBM-SPSS version 24.0. All values are given as (mean ± SD) unless specified. Sensitivity (true positive/(true positive + false negative)), specificity (true negative/true negative + false positive), positive predictive value (true positive/(true positive + false positive)) and negative predictive value (true negative/(true negative + false negative)) were calculated accordingly. It was suggested from a meta-analysis of the literature that an AMH greater than 35 pmol/l was suggestive of a diagnosis of PCOS^6^ and therefore this value was used rather than the higher cut off of 55 pmol/l recently suggested^12^. A biochemical diagnosis of PCOS was defined as requiring an AMH >35 pmol/l and a raised androgen (true positive), whilst the exclusion of PCOS was defined as an AMH <35 pmol/l and a normal androgen level in the control population (true negative). A false negative was defined as either having one of an AMH >35 pmol/l or an elevated androgen level in the PCOS population; a false positive was defined as an AMH >35 pmol/l and an elevated androgen in the control population.

## Results

**Figure 1:**
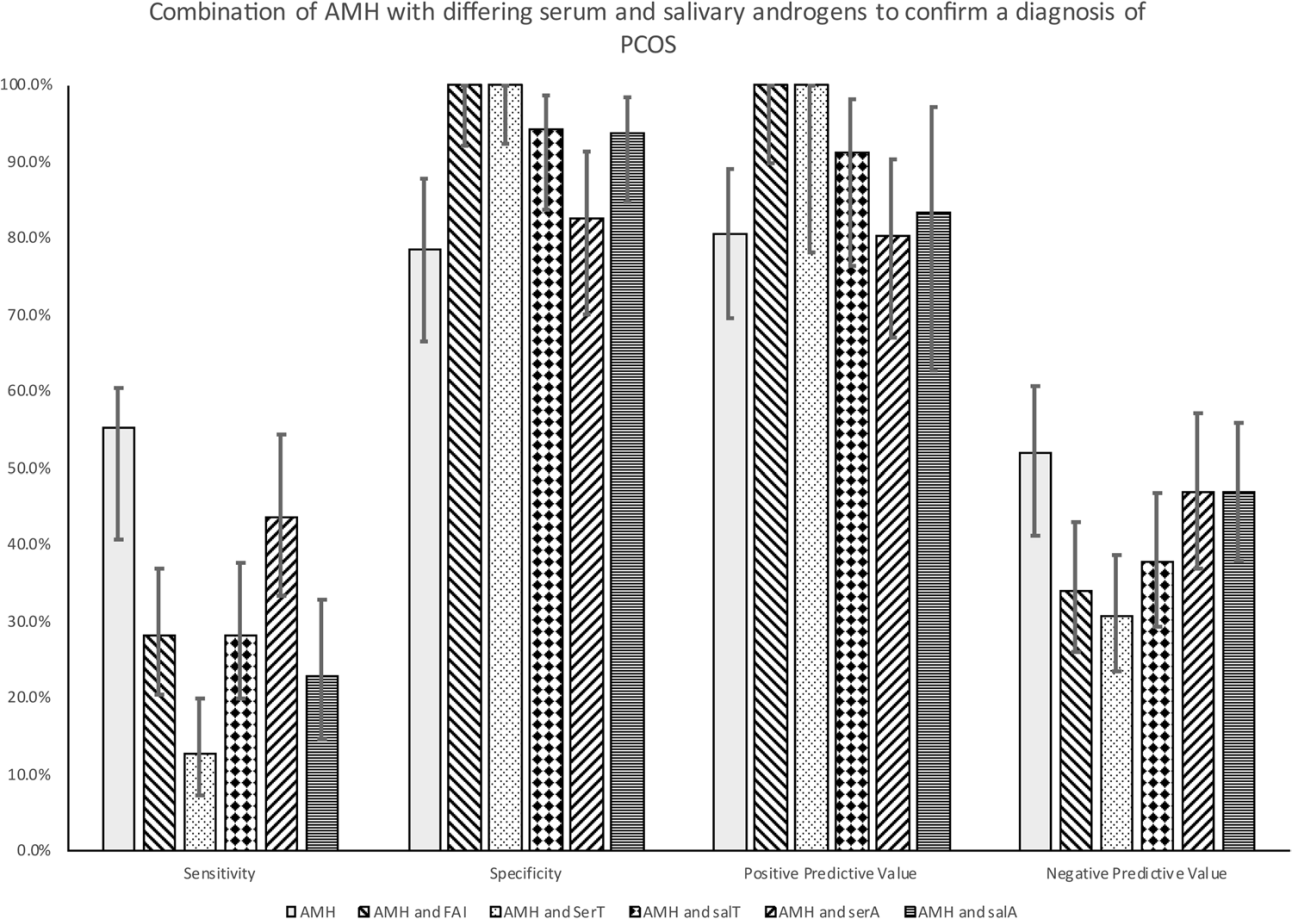
Sensitivity, specificity, positive predictive values and negative predicted values for a biochemical diagnosis of PCOS based on an anti-Müllerian hormone (AMH) measurement (greater or less than 35 pmol/l) with, calculated free androgen index (FAI, greater or less than 4); serum testosterone (greater or less than 2 nmol/l); salivary testosterone (salT, greater or less than 19 pmol/l); serum androstenedione (greater or less than 6 nmol/l); salivary androstenedione (greater or less than 250 pmol/l). Participants with polycystic ovary syndrome (n = 105) and normal controls (n = 65).

All PCOS women were categorized into a single phenotype according to the combination of the three Rotterdam’s Consensus Criteria. The baseline demographics of patients have been reported for this cohort^7^ (Table 1). As expected, patients with PCOS showed higher insulin levels, HOMA-IR, 2 hour glucose post oral glucose tolerance test (OGTT) values (p < 0.001) (Table 1). Serum T, FAI, A and salT were significantly elevated in PCOS compared to controls (p < 0.001). The sensitivity, specificity, positive and negative predictive values for AMH alone were 55%, 78%, 80% and 52%. The sensitivity, specificity, positive and negative predictive values for AMH and T, FAI and salT are shown in Fig. 1. The combination of a raised AMH with either a raised serum T or raised FAI was 100% specific for a diagnosis of PCOS in this population with a positive predictive value of 100%; this was less robust for the salT with a specificity of 94% and a positive predictive value of 91%. Conversely, the combination of an AMH <35 pmol/l with either a normal T or FAI was 100% specific and with a 100% positive predictive value for the exclusion of a diagnosis of PCOS. The sensitivity, specificity, positive and negative predictive values for AMH and A and salT are shown in Fig. 1. The combination of a raised AMH with a raised serum A, or a raised AMH and a raised salA gave a specificity of 87% and 94%, respectively; a sensitivity of 43% and 23%, respectively, for a diagnosis of PCOS in this population, with positive and negative predictive values being correspondingly low.

**Table 1:** Comparison of anthropometric and hormonal parameters of the 170 subjects involved in the study, 65 women without PCOS and 105 women with PCOS. The diagnosis of PCOS was based on all three diagnostic criteria of the Rotterdam consensus, namely clinical and biochemical evidence of hyperandrogenism (Ferriman-Gallwey score >8; free androgen index >4 respectively), oligomenorrhea or amenorrhea and polycystic ovaries on transvaginal ultrasound. These women therefore represented the phenotype with the greatest metabolic features.

Parameters	Controls		PCOS		p value
	Median	IQR	Median	IQR	
Age (years)	31.0	11.0	27.0	11.0	0.010*
BMI (kg/m^2^)	25.0	6.1	33.0	10.2	<0.001*
Waist Circumference (cm)	78.0	14.8	101.0	21.0	<0.001*
Hip Circumference (cm)	100.0	16.0	117.0	19.5	<0.001*
AMH (pmol/l)	18.1	24.8	40.0	42.7	<0.001*
Salivary testosterone (pmol/l)	13.1	10.0	18.5	15.0	<0.001*
Total Testosterone (nmol/L)	1.0	0.5	1.3	0.9	<0.001*
Salivary Androstenedione (pmol/l)	142.89	95.00	165.76	118.00	<0.001*
Androstenedione (nmol/L)	7.40	5.90	40.31	7.90	<0.001*
SHBG (nmol/L)	45.0	31.8	27.0	19.0	<0.001*
FAI (%)	2.2	1.9	4.5	5.3	<0.001*
FSH (IU/L)	5.6	3.6	4.9	2.8	0.099
LH (IU/L)	4.3	5.4	6.1	5.5	0.009*
Baseline Glucose (mmol/L)	4.5	0.6	4.7	0.5	0.001*
2 Hour Glucose (mmol/L)	4.9	1.3	5.6	1.8	<0.001*
Insulin (μIU/ml)	6.0	3.8	13.7	11.4	<0.001*
HOMA-IR	1.2	0.8	2.9	2.4	<0.001*

BMI – Body Mass Index; AMH- antiMullarian hormone; SHBG- sex hormone binding globulin; FAI–Free Androgen Index; FSH–Follicle Stimulating Hormone, LH–Leutenising hormone; HOMA-IR–Homeostasis model of assessment–insulin resistance

To convert values for glucose to milligrams per deciliter, divide by 0.056.

To convert values for insulin to picomoles per liter, multiply by 6.

To convert values for testosterone to nanograms per deciliter, divide by 0.03467.

To convert values for SHBG to micrograms per deciliter, divide by 34.7.

## Discussion

This study shows an AMH above a conservative threshold of 35 pmol/l with either a raised T or a raised FAI identified 100% of PCOS women with type A phenotype with a 100% positive predictive value, though sensitivity was low. Conversely, but equally important, the combination of a an AMH less than 35 pmol/l with a normal T or normal salT excluded a diagnosis of PCOS with 100% specificity, but again the sensitivity was low. AMH with salT showed high specificity but the salT identified 3 control patients with an elevated level salT and AMH making an apparent false positive, though this would have been addressed by a higher AMH cut off of 46 pmol/l (or higher)^12^. SalT correlates very closely with FAI and it would have been predicted that both would have had equal utility in combination with AMH^7^, though in this regard FAI would appear to be the better androgen to measure. This would appear to be the first time that a combination of 2 biochemical markers have shown 100% specificity for the diagnosis or the exclusion of PCOS, though this needs to be validated in a larger cohort and with the other phenotypes identified by the Rotterdam criteria, other than the single phenotype here.

The Rotterdam criteria are the most commonly used to make the diagnosis of PCOS but there is controversy in making a diagnosis of PCOS in adolescence and the perimenopause^3^, whilst the accuracy of androgen measurements^13^ and the polycystic ovarian morphology^14^ may also contribute to diagnostic uncertainty for any given case. In addition, the diagnosis of PCOS can only be made once other causes have been excluded^3^; therefore, a biomarker or combination of biomarkers to allow a positive diagnosis of PCOS is of major interest to help make a positive diagnosis of PCOS and to reduce the uncertainty that may result for individual patients; AMH has been suggested to be such a biomarker^6,12^. AMH at a cut off of 55 pmol/l has been reported to have a high specificity and sensitivity and was robust for PCOS in older age groups^12^. A lower threshold of 35 pmol/l was reported to be of value in a meta-analysis; the specificity shown here was similar to the meta-analysis was similar (78% v 82%) though the sensitivity was less (55% v 79%), perhaps due to the different PCOS populations studied. These reports show the lack of standardisation and variability in the AMH assays that would result in differing cut off thresholds^1,15^. This also highlights that there is not an AMH threshold cut off level that is diagnostic, in the absence of other factors, to positively make a 100% diagnosis of PCOS, nor to positively exclude the diagnosis. It is not clear how an AMH level should be incorporated into a PCOS diagnostic algorithm.

Positive aspects of this study included the significant number of subjects all with the type A phenotype for PCOS, fulfilling all 3 diagnostic criteria, and a homogeneous Caucasian population; AMH is noted to be the highest in this PCOS population^5,16^. The utility of this approach using only PCOS subjects with phenotype A was that if the AMH/androgen combination had not shown such specificity and positive predictive values then it would have certainly been less for the other PCOS phenotypes, and would therefore have no diagnostic discriminatory value. All patients had polycystic ovaries on ultrasound and therefore it might have expected all of the AMH values to be elevated. Why this was not the case is likely due to the observation that AMH values have been shown to be lower in anovulation and many of these patients may have been anovulatory, though this was not determined in this study, and AMH levels differ with age^12^.

The combination of AMH with either serum A or salA gave specificities that were quite high but the sensitivities were low. It has been suggested that A may reflect hyperandrogenism better than serum T^8^ and it has been suggested to be of diagnostic utility in European guidelines^9^; however, in combination with AMH, this study suggests that it may not be useful in making a diagnosis of PCOS with type A phenotype.

Limitations of this study were, in part, the converse of the strengths. This was a narrow group of individuals all with the type A PCOS phenotype, and therefore it is not known if an AMH >35 pmol/l with a raised FAI would be 100% diagnostic for the B, C and D phenotypes. In addition, this was a narrow ethnic group and therefore more detailed studies of the different PCOS phenotypes with other ethnicities whose PCOS prevalence may differ, are required.

In conclusion, the combination of a raised AMH >35 pmol/l and a raised FAI is 100% specific for a diagnosis of PCOS with type A phenotype and conversely a AMH <35 pmol/l with a normal FAI was 100% specific for the exclusion of PCOS with type A phenotype, though sensitivity was poor; thus, the additional routine measurement of AMH may be of value that would help in cases of diagnostic uncertainty for PCOS with type A phenotype.
